# Rapid and High-Throughput *pan-Orthopoxvirus* Detection and Identification using PCR and Mass Spectrometry

**DOI:** 10.1371/journal.pone.0006342

**Published:** 2009-07-22

**Authors:** Mark W. Eshoo, Chris A. Whitehouse, Aysegul Nalca, Scott Zoll, Joseph A. Ecker, Thomas A. Hall, Thuy-Trang D. Pennella, David D. Duncan, Anjali Desai, Emily K. Moradi, Karl Rudnick, Brian Libby, Raymond Ranken, Rangarajan Sampath, Steven A. Hofstadler, David J. Ecker, Lawrence B. Blyn

**Affiliations:** 1 Ibis Biosciences, Carlsbad, California, United States of America; 2 United States Army Medical Research Institute of Infectious Diseases, Fort Detrick, Maryland, United States of America; 3 Science Applications International Corporation (SAIC), San Diego, California, United States of America; Institute of Molecular and Cell Biology, Singapore

## Abstract

The genus *Orthopoxvirus* contains several species of related viruses, including the causative agent of smallpox (*Variola* virus). In addition to smallpox, several other members of the genus are capable of causing human infection, including monkeypox, cowpox, and other zoonotic rodent-borne poxviruses. Therefore, a single assay that can accurately identify all orthopoxviruses could provide a valuable tool for rapid broad orthopovirus identification. We have developed a *pan-Orthopoxvirus* assay for identification of all members of the genus based on four PCR reactions targeting *Orthopoxvirus* DNA and RNA helicase and polymerase genes. The amplicons are detected using electrospray ionization-mass spectrometry (PCR/ESI-MS) on the Ibis T5000 system. We demonstrate that the assay can detect and identify a diverse collection of orthopoxviruses, provide sub-species information and characterize viruses from the blood of rabbitpox infected rabbits. The assay is sensitive at the stochastic limit of PCR and detected virus in blood containing approximately six plaque-forming units per milliliter from a rabbitpox virus-infected rabbit.

## Introduction

The orthopoxviruses (family *Poxviridae*, genus *Orthopoxvirus*) are a diverse group of large, enveloped viruses that contain a covalently closed, double-stranded DNA genome of approximately 200 kbp [1]. The genus is comprised of at least 10 recognized species. Several viruses within this group are significant human pathogens, including *Variola* virus (the causative agent of smallpox), monkeypox virus, cowpox virus, and *Vaccinia* virus. Other members, including raccoonpox, camelpox, ectromelia (mousepox), taterapox, and volepox viruses have only been isolated from their respective mammalian hosts. Although many orthopoxviruses specifically infect certain animal hosts, others (e.g., monkeypox and cowpox viruses) can also infect humans and are considered zoonotic pathogens. In humans, symptoms of orthopoxvirus infections range from mild skin lesions to fatal systemic disease. For example, smallpox produced a generalized rash that progressed from the papular to vesicular to pustular stages and resulted in a greater than 30% mortality rate in unvaccinated persons [2]. Although naturally-occurring smallpox was eradicated nearly three decades ago [3], official stocks of the virus still remain in two locations, one at the U.S. Centers for Disease Control and Prevention in Atlanta, GA and the other at the State Research Center of Virology and Biotechnology, Novosibirsk, Russia. This, in addition to waning immunity against smallpox within the human population, has led to concerns that *Variola* virus might be used as a bioweapon [4].

Monkeypox virus causes a disease similar to smallpox in humans, but results in a lower fatality rate [5]. Monkeypox virus is primarily transmitted to humans through direct contact with infected animals, generally various species of rodents or squirrels in the rain forests of central Africa. However, additional attention was brought to bear on this virus when, in the spring of 2003, it emerged for the first time in the Western Hemisphere and caused a cluster of cases in the U.S. Midwest [6].

*Vaccinia* virus is famous for being the vaccine that was used to eradicate smallpox. It was also the first animal virus to be purified and chemically analyzed and was the first to be genetically engineered [7]. Despite its notoriety, however, its origin and natural history remain obscure. Recent evidence suggests that *Vaccinia* virus and horsepox virus are very similar phylogenetically and share a relatively recent common ancestor [8]. *Vaccinia* virus is often confused with cowpox virus; although it is now well established that they are distinct virus species [9]. In fact, cowpox virus is considered a zoonotic pathogen and is seen in a broad range of host species, most notable wild rodents, but rarely in cattle, as its name would imply. Interestingly, wild and domestic cats and elephants appear to be highly susceptible to infection with cowpox virus [9]. Numerous recent studies have uncovered several novel vaccinia-like viruses that have caused zoonotic outbreaks in Brazil [10], [11], [12], [13], [14], [15], [16]. It is likely that as various ecological niches are examined further, even more species of *Orthopoxvirus* will be identified.

Several genus-specific assays have been described for the detecting and discriminating various *Orthopoxvirus* members that require conventional PCR followed by restriction endonuclease digestion and subsequent gel electrophoresis [17], [18]. More recently, several real-time LightCycler-based PCR assays have been developed for *pan-Orthopoxvirus* or specific orthopoxvirus species detection [19], [20], [21]. For these assays, differentiation of the various orthopoxvirus species requires the use of different TaqMan probes in separate reactions or melt-curve analysis of hybridization probes. Here, we describe a rapid, high-throughput, multi-locus method for identifying orthopoxviruses based on PCR amplification followed by electrospray ionization mass spectrometry (PCR/ESI-MS) performed on the Ibis T5000 instrument [22], [23], [24], [25]. This technology has been applied to detection and identification of other viral pathogens, including alphaviruses [26], influenza viruses [27], adenovirus [28], and coronaviruses [29]. The assay described here is extremely sensitive and able to detect and identify each species from a diverse collection of orthopoxviruses.

## Results

### Orthopoxvirus PCR primer pairs

**Figure 1:**
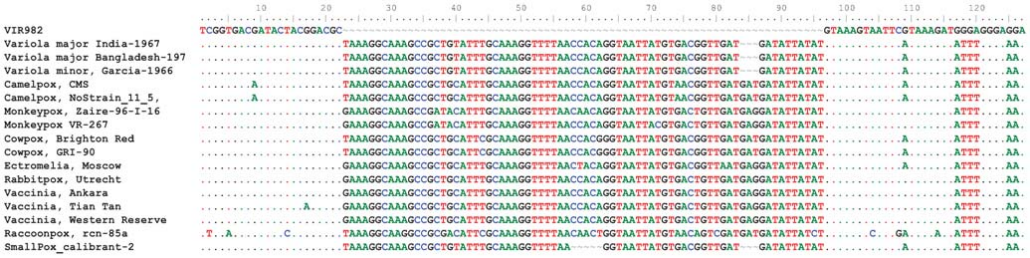
Alignment of Orthopoxvirus sequences showing conservation of PCR primer VIR982 within this viral family, flanking a region of species-specific variations. “Dots” in a column represent homology to the reference sequence above. The primer 5 prime sequence shows the addition of non-homlogous nucleotides to increase the Tm of the primers after the first PCR cycles.

The genus *Orthopoxvirus* consists of least 10 species. The goal of this work was to develop a simple assay that would allow the detection of all of the diverse members of this group of viruses. Several sets of primers were evaluated for their analytical sensitivity, *pan-Orthpoxvirus* coverage and performance under uniform PCR conditions. Four primer pairs were chosen for use in the final assay format (Table 1). Several of the primers have non-homologous nucleotides at their 5′ ends to raise the temperature of melting (Tm) of the primers after the first round of amplification as orthopoxviruses have a low GC genome. Primer Pair VIR982 exhibited the greatest analytical sensitivity of the four primers in the assay. Figure 1 shows the alignment of this primer pair against the known sequences of several diverse orthopoxviruses. For each primer region, a database of expected base compositions (A, G, C, and T base counts) from all known orthopoxvirus sequences in GenBank was generated (data not shown) and used in the identification and classification of the test isolates.

**Table 1:** PCR primers used in this study.

Primer pair ID	Primer code	Gene target	Primer sequence (5′-3′)	3′ nt position*
VIR982	VIR2545F	DNA polymerase	TCGGTGACGATACTACGGACGC	46717
	VIR2546R		TCCTCCCTCCCATCTTTACGAATTACTTTAC	46645
VIR985	VIR2550F	RNA helicase	TGGAAAGTATCTCCTCCATCACTAGGAAAACC	58378
	VIR2551R		TCCCTCCCTCCCTATAACATTCAAAGCTTATTG	58426
VIR979	VIR2539F	DNA helicase	TGATTTCGTAGAAGTTGAACCGGGATCA	117490
	VIR2540R		TCGCGATTTTATTATCGGTCGTTGTTAATGT	117560
VIR988	VIR2556F	RNA polymerase	TCCTCCTCGCGATAATAGATAGTGCTAAACG	123174
	VIR2557R		TGTGTTCAGCTTCCACCAGGTCATTAA	123216

* Position of 3′ nucleotide against reference genome: Variola major, Syria 1972, DQ437592.

Based upon the predicted and experimentally determined amplicons, these primers effectively resolve all known *Orthopoxvirus* species from one another. All experimentally determined base-count signatures were found to agree with bioinformatically predicted signatures based upon published genomic sequences.

### Detection of diverse orthopoxvirus isolates

**Figure 2:**
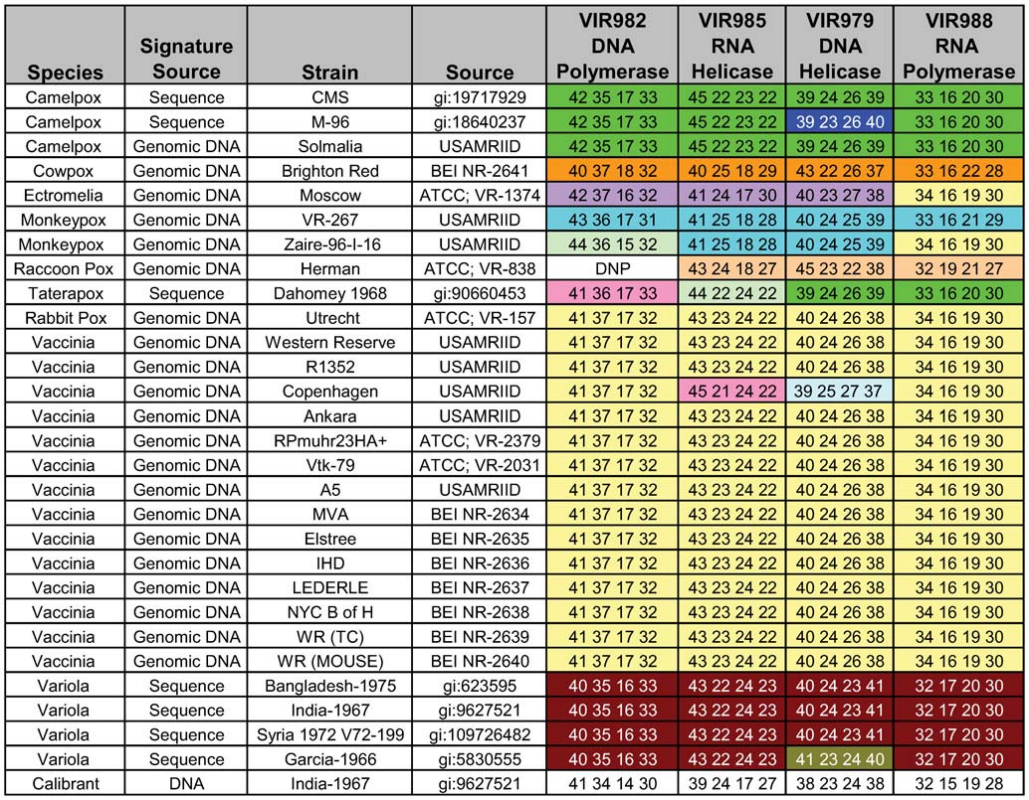
Base compositions of the PCR amplicons generated using primer pairs VIR982, VIR985, VIR979, and VIR988. Within each column, identical base compositions for different isolates for a particular primer pair are shown grouped by the same color. DNP (did not prime) indicates that a PCR amplicon was not generated using the specified primer pair and viral DNA. The numbers in the columns under the primer pair ID indicates the numbers of each base (A, G, C, and T) in the PCR amplicons generated from the target virus. Signature source indicates whether the base composition signatures were determined from sequence or experimentally using viral genomic DNA.

As shown in Figure 2, all of the four primer pairs produce amplicon that can distinguish *Variola* virus from any other orthopoxvirus. Primer pair VIR979 could resolve *Variola major* virus from *Variola minor* virus and camelpox strain CMS from strain M-96. Primer pairs VIR982, VIR985 and VIR988 could resolve the two strains of monkeypox tested: VR-267 and Zaire-96-1-16. All of the *vaccinia* isolates tested including rabbitpox and horsepox, which are sub-species of *vaccinia*, have a common base count signature for primer pair VIR9888 ( 34A, 16G, 19C, 30T). *Vaccinia*, Copenhagen strain and horsepox (gi|111184167) can be distinguished from each other and other *vaccinia* isolates based upon their unique base-count signatures for primers pairs VIR985 and VIR979.

In addition to providing sub-species resolution, the use of four PCR primers in the assay enabled the detection and identification of an orthopoxvirus at the stochastic limit of PCR: 4–8 copies/PCR reaction. For example if only 4–8 genomes/PCR are used in the assay there is a very high probability that at least one of the four primers would detect the virus and provide information sufficient for its identification.

The assay is specific to Orthopoxvirus. Nucleic acid extracts from the blood of non-infected rabbits (N = 4) and humans (20 ng and 500 ng/PCR reaction) failed to produce an amplicon other than the internal positive controls. As expected, swinepox, a suipoxvirus (ATCC VR-363), in the family Poxviridae failed produce an amplicon other than the internal positive controls using the assay. We further tested a panel of DNA viruses in the assay to further define specificity including HSV1, adenovirus types 1, 5, 8, 4, 7A, varicella zoster virus (VZV), HPV16 & 18, human parvo virus B19, BK virus and JC virus. None of these viruses cross-reacted using the assay.

### Analytical sensitivity and detection of virus in biological samples

To test the analytical sensitivity of the assay for *Variola* virus we performed a dilution to extinction using the *Variola* virus derived calibrant. The calibrant was quantified by OD_260_ and diluted down to determine the limit of detection by the assay. The calibrant is present in every PCR reaction at 100 copies.

**Figure 3:**
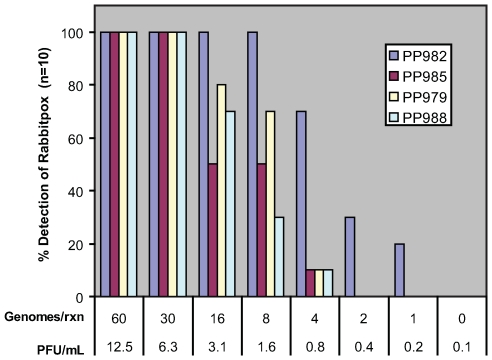
Analytical sensitivity of PCR primer pairs VIR982, VIR985, VIR979, and VIR988 in amplification of an extract from rabbitpox-infected rabbit blood (sample ID; 3E-day 6). Ten replicate detections were performed for each dilution and a score of 100% indicates that viral-derived amplicon was detected in each of ten replicates.

Blood extract from rabbitpox-infected rabbits was taken six days after exposure to aerosolized rabbitpox virus. The first blood sample evaluated (sample ID: 3E-day 6) was found to have 4×10^3^ plaque forming units (PFU) per mL of blood as determined by a plaque assay on Vero cells. DNA extract from this sample was analyzed by real-time PCR and found to contain 4×10^6^ genomes/mL of blood, assuming 100% extraction efficiency. Blood taken on the 6^th^ day post infection from a second infected rabbit (sample ID: 3D-day6) had 3.3×10^2^ PFU/mL blood and 2.5×10^5^ genomes/mL blood. The most sensitive primer pair, VIR985, detected eight genomic copies of rabbitpox genome isolated from infected rabbit blood with 100% efficiency in 10 out 10 PCR replicates, which is near or at the stochastic limits of PCR detection. The viral PFU in this blood samples was determined prior to DNA extraction and the diluted DNA extract would equate to less than 2 PFU/mL of blood. The three additional primer pairs are sensitive to 30 genomes per PCR reaction, which corresponds to a detection of 6.3 PFU/mL of blood, with 100% detection in 10 out 10 PCR replicates and provide added sub-species information and redundancy to the assay (Figure 3).

## Discussion

Today, orthopoxviruses are principally rare zoonotic pathogens, but historically *Variola* was a devastating human pathogen [30]. Though *Variola* virus has been eradicated there is still the potential for its reintroduction into the human population through an act of bioterrorism. It is also possible that a zoonotic orthopoxvirus could emerge as a *variola*-like virus of humans or be used as a bioterrorist agent. A single assay that can detect and identify all orthopoxviruses is critical for effective surveillance. Though several methods exist for the detection and surveillance of orthopoxvirus, these assays are limited in their coverage or require large numbers of reactions to identify all *Orthopoxvirus* species. In this study, we describe an assay using four PCR primer pairs that can identify and distinguish all orthopox species and can provide subspecies level of identification of several important orthopoxviruses. Our primers target the highly conserved core viral DNA and RNA polymerase and helicase genes and thus it is very likely that novel orthopox viruses will also be detected using this strategy.

In this study we tested the assay on a diverse panel of orthopoxviruses and also tested detection of rabbitpox from infected rabbits. Rabbitpox is a vaccinia virus but does not infect humans and therefore is an ideal model for studying human smallpox [31]. There was a 1,000-fold difference between PFU and genome levels from the blood samples studied; this also was true for several samples from different animals and probably represents a high level of non-viable or replicating virus in the blood. Similarly high genome to PFU ratios was observed for other viruses [32].

Overall we demonstrated that the *pan-Orthopoxvirus* assay can detect a wide and diverse range of orthopoxvirus and can be used to accurately speciate orthopoxvirus. The assay is simple and can be performed using premade PCR plates that contain all reaction components except genome. After extraction, the samples can be processed from PCR through analysis and reporting in five hours. Using the described assay, a single 96 well PCR plate can be used to analyze 24 samples and the system can process 16 plates in a 24 hour period for a total throughput of 384 samples per day on a single instrument. Such throughput is essential for surveillance and in times of an epidemic outbreak or acts of bioterrorism.

## Materials and Methods

### Viral isolates and DNA extraction

The viral strains used in this study were provided by the U.S. Army Medical Research Institute of Infectious Diseases (USAMRIID), the American type culture collection (ATCC), or the Biodefense and Emerging Infections Research Resources Repository (BEI Resources) and are listed in Table 2. Samples from BEI resources were supplied as DNA extracts. DNA from viral isolates from USAMRIID and ATCC were extracted using the QiaAmp MinElute Virus spin kit (Third Edition, February 2007 version) according to the manufacturers recommended procedure (Qiagen Inc, Valencia, CA).

**Table 2:** Orthopoxvirus species/strains used in this study.

Virus species	Strain	Year of isolation	Origin	Source
*Camelpox virus*	903	1978	Somalia	USAMRIID
*Cowpox virus*	Brighton Red	1938	Great Britain	BEI NR-2641
*Ectromelia virus*	Moscow	?	?	ATCC; VR-1374
*Monkeypox virus*	VR-267	1962	West Africa	USAMRIID
	Zaire-96-I-16	1996	Zaire, Africa	USAMRIID
*Rabbitpox virus*	Utrecht	1941	Holland	ATCC; VR-157
*Raccoonpox virus*	Herman	1964	Maryland, USA	ATCC; VR-838
*Vaccinia virus*	Western Reserve	1941	Cleveland, Ohio, USA	BEI NR-2640
	R1352	?	?	USAMRIID
	Copenhagen	?	Denmark	USAMRIID
	Ankara	1954	Ankara, Turkey	USAMRIID
	RPmuhr23HA+	1993	Florida, USA	ATCC; VR-2379
	Vtk-79	?	?	ATCC; VR-2031
	A5	?	?	USAMRIID
	Modified Vaccinia Ankara (MVA)	1971	Munich, Germany	BEI NR-2634
	Lister	1892	Elsetree, London	BEI NR-2635
	IHD	1954	New York City Department of Health	BEI NR-2636
	Lederle	?	?	BEI NR-2637
	NYCBH	1856	England	BEI NR-2638

### Rabbitpox virus-infected rabbits

Viral extracts from rabbitpox virus-infected rabbits were obtained from USAMRIID. Rabbits were bled every other day after challenge with aerosolized rabbitpox virus. Blood was collected into EDTA tubes and 100 µL of blood was used to isolate viral DNA with the BioRobot M48 (Qiagen) using the QIAamp blood kit (Qiagen version February 2003) in accordance with manufacturer's instructions. Real-time PCR was carried out with the LightCycler (Roche, Indianapolis, IN) using a *pan-Orthopoxvirus* assay as previously described [20]. Briefly, the oligonucleotide primers and a minor groove binder (MGB) protein-containing TaqMan probe were designed to hybridize to conserved regions of the orthopoxvirus hemagglutinin (HA) gene; sequences have been published elsewhere [20]. Reactions were performed on a Roche LightCycler. Virus was quantified using standards based on the cloned Orthopoxvirus HA (J7R) gene were calculated using the LightCycler software version 4.0 as described by Kulesh et al[33]. Viral titers were determined by plaque assay on Vero cells grown in Earle's modified Eagle's medium supplemented with 10% fetal calf serum.

### Primer design

Four primer pairs were designed to target the DNA and RNA polymerase genes and the DNA and RNA helicase genes in the conserved core region of viruses within the family *Poxviridae* (Table 1). Each of these primers generated an amplicon from any orthopoxvirus DNA template tested. All primers used in this study had a thymine nucleotide at the 5′-end to minimize addition of non-templated adenosines during amplification using Taq polymerase [34].

### PCR

The PCR reaction mix consisted of 2.4 U of Faststart Taq polymerase (Roche Applied Science, Indianapolis, IN), 20 mM Tris (pH 8.3), 75 mM KCl; 1.5 mM MgCl_2_; 0.4 M betaine; 800 µM equal mix of dCTP, dTTP, dGTP, and dATP; and 250 nM of each primer. The following PCR cycling conditions were used on MJ dyad 96-well thermocyclers (Bio-Rad Inc. Hercules, CA): 95°C for 10 min, followed by 8 cycles of 95°C for 30 s, 48°C for 30 s, and 72°C 30 s, with the 48°C annealing temperature increasing 0.9°C each cycle. The PCR was then continued for 37 additional cycles of 95°C for 15 s, 56°C for 20 s, and 72°C for 20 s. The PCR cycle ended with a final extension of 2 min at 72°C followed by a 4°C hold.

### Internal positive control DNA

An internal positive control (calibrant) made from synthetic DNA (BlueHeron Biotechnology, Bothell, WA) was included in each PCR reaction at an experimentally determined concentration (100 copies/PCR reaction). The calibrant was based upon the primer target region of *Variola major virus* (gi|9627521) and served as our test surrogate for this virus. The calibrant sequence contains a five-base pair (bp) deletion within the amplicon so that the calibrant amplicons could readily be resolved from the viral template-derived amplicon.

### Mass spectrometry and base composition analysis

After amplification, 30 µL aliquots of each PCR reaction were desalted and purified by using a weak anion exchange protocol described elsewhere. [22]. Accurate mass (±1 ppm), high-resolution (M/dM>100,000 FWHM) mass spectra were acquired for each sample using high-throughput ESI-MS protocols described previously [24]. For each sample, approximately 1.5 µL of analyte solution was consumed during the 74-second spectral acquisition. Raw mass spectra were post-calibrated with an internal mass standard and deconvolved to monoisotopic molecular masses. Unambiguous base compositions were derived from the exact mass measurements of the complementary single-stranded oligonucleotides [35] . Quantitative results were obtained by comparing the peak heights with the calibrant present in every PCR well at 100 molecules as previously described for other Ibis T5000 assays [24].
